# Laceration of the transverse mesocolon in an old man with a habit of abdominal massage for constipation: a case report

**DOI:** 10.1186/s40792-019-0767-6

**Published:** 2020-01-02

**Authors:** Shusuke Mori, Tomohiko Ai, Yasuhiro Otomo

**Affiliations:** Trauma and Acute Critical Care Center, Tokyo Medical and Dental University, 1-5-45 Yushima, Bunkyo-ku, Tokyo, 113-8519 Japan

**Keywords:** Blunt abdominal trauma, Constipation, Abdominal massage, Mesenteric laceration

## Abstract

**Background:**

Abdominal massage for the resolution of constipation has been reported to be safe and recommended in some studies. It is conventionally performed for the elderly suffering from intractable constipation. Meantime, isolated mesenteric injury after blunt abdominal trauma is uncommon. Here, we report a case of isolated mesenteric injury following self-abdominal massage for constipation.

**Case presentation:**

A 68-year-old man consulted a local hospital due to a sudden abdominal pain. He had a history of prostate cancer treated with radiation therapy 3 years ago, and he had been suffering from chronic constipation for many years. A plain computed tomography (CT) revealed a fist-sized homogeneous mass-like lesion located in the left upper abdomen and a moderate amount of ascites. With the initial diagnosis of a malignant tumor accompanied by peritonitis carcinomatosa, he was hospitalized for further examinations. On the next day, his hemoglobin concentration dropped from 11.6 to 6.6 g/dl, and diagnostic paracentesis showed bloody ascites. He was urgently transferred to our tertiary emergency center. An enhanced CT demonstrated a non-enhanced, homogeneous, 8.5 cm in diameter, mass lesion located to the posterior of the stomach with massive bloody ascites. He underwent an emergency exploratory laparotomy, and it showed a 5 cm of laceration in the transverse mesocolon adjacent to Treitz’s ligament and approximately 1.5 l of intraabdominal hemorrhage. Hemostasis of the bleeding from the laceration was achieved by suture ligations, but the gap of the laceration could not be closed by suturing because the tissue was too fragile. Blood transfusion with 4 units of packed red blood cells and 10 units of fresh frozen plasma was performed during operation. He was discharged without any significant complications except for postoperative paralytic ileus. Later on, it turned out that he had a habit of massaging his abdomen for the resolution of intractable constipation and did it hard 1 day before the onset.

**Conclusions:**

This is the first report of life-threatening mesenteric injury caused by self-abdominal massage to resolve constipation, though other etiologies such as rupture of small aneurysms could not perfectly be excluded. Abdominal massage is reported to be effective and safe for the resolution of constipation; however, this case demonstrated it could be detrimental.

## Introduction

Isolated mesenteric injury is uncommon, though the intestine and the mesentery are the third most common injured organs caused by blunt abdominal trauma [1]. The mesentery is morphologically a fan-shaped structure arising from the root of the superior and inferior mesenteric arteries and veins, and it consists of the branches of these vessels and soft connective tissues. This anatomical feature makes the mesentery vulnerable to external forces.

Constipation is one of the most common disorders seen in the elderly. It is known to cause uncomfortable symptoms and a variety of medical problems. To avoid aggravation of constipation, preventative measures such as laxatives and enemas are often taken in the clinical settings [2]. Interestingly, it has been reported that abdominal massage improves bowel habits in the elderly, and no serious complications have been described [3]. Though it seems easy to imagine that excessive abdominal massage may lead to organ injuries, such risks have seldom been pointed out [4]. Here, we present the first case report of life-threatening mesenteric injury with massive intraabdominal hemorrhage caused by self-abdominal massage to resolve constipation.

## Case presentation

**Fig. 1:**
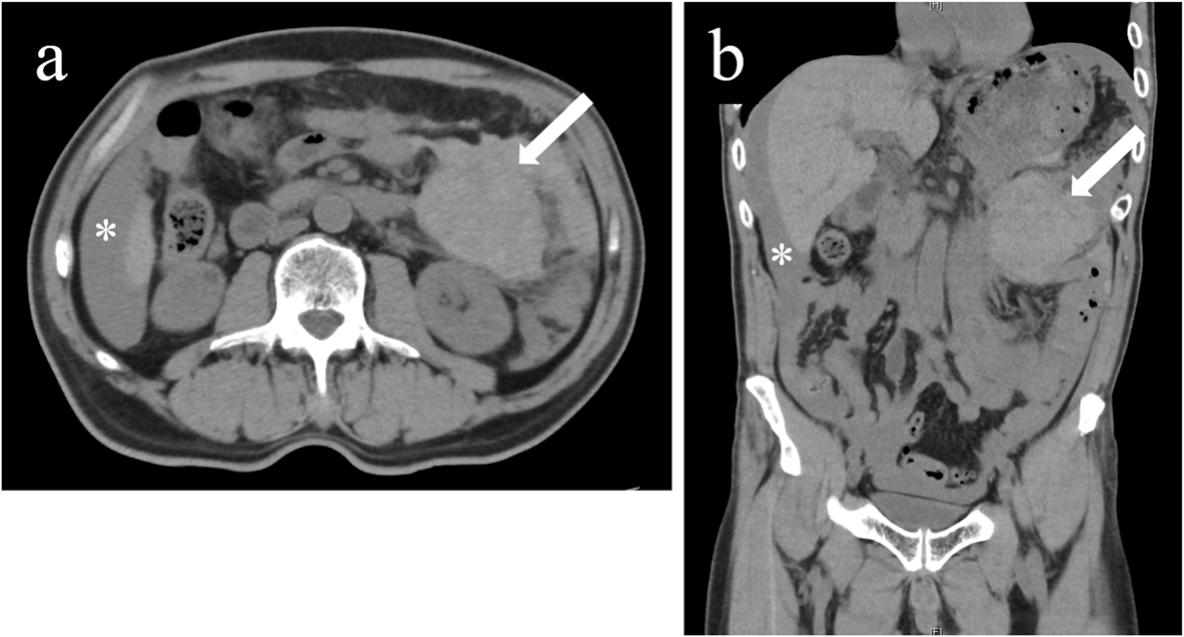
A computed tomography without contrast performed in the former hospital is shown; **a** an axial section and **b** a coronal section. A homogeneous mass lesion is located between the posterior wall of the stomach and the anterior side of the pancreatic tail (arrows) with intra-abdominal fluid collection (asterisks)

A 68-year-old man with chronic constipation visited a nearby hospital complaining of sudden-onset upper abdominal pain. His past medical history was significant for prostate cancer treated with radiation therapy 3 years ago. He also underwent endoscopic polypectomy for colonic polyps a year ago. At the initial evaluation, he was totally conscious but in a shock state with his heart rate 100 beats per minute, blood pressure 65/39 mmHg, respiratory rate 20 times per minute, body temperature 36.6 °C, and oxygen saturation 98% in room air. He responded to fluid resuscitation. A computed tomography (CT) without contrast demonstrated a moderate amount of ascites and a fist-sized, homogeneous, mass lesion located between the stomach and the tail of the pancreas (Fig. 1). Considering his past medical history, he was initially diagnosed to have a malignant tumor with peritonitis carcinomatosa, and he was hospitalized for further examinations and observation. On the next day, his hemoglobin level unexpectedly dropped from 11.1 to 6.6 g/dl. In addition, his diagnostic abdominal paracentesis showed a collection of bloody fluids which indicated intra-abdominal hemorrhage, and he was transferred to our hospital, a tertiary emergency center, for further diagnosis and treatments.

On arrival at our hospital, he was alert and well oriented. His vital signs were as follows: heart rate 116 beats per minute, blood pressure 114/71 mmHg, respiratory rate 18 times per minute, body temperature 36.6 °C, and oxygen saturation 98% in room air. A physical examination revealed moderate tenderness in his left upper abdomen with a peritoneal sign. He did not show any traumatic findings. His electrocardiogram showed sinus tachycardia without other abnormalities. A chest X-ray revealed normal findings. His laboratory data showed mild leukocytosis (white blood cell 11.5 × 103/μL), moderate anemia (hemoglobin 6.6 g/dL), elevated urea nitrogen level (34 mg/dL), and high serum amylase (812 U/L). His venous blood gas result revealed his pH 7.436, HCO_3_^-^ 28.1 mmol/L, and lactic acid 1.2 mmol/L.

**Fig. 2:**
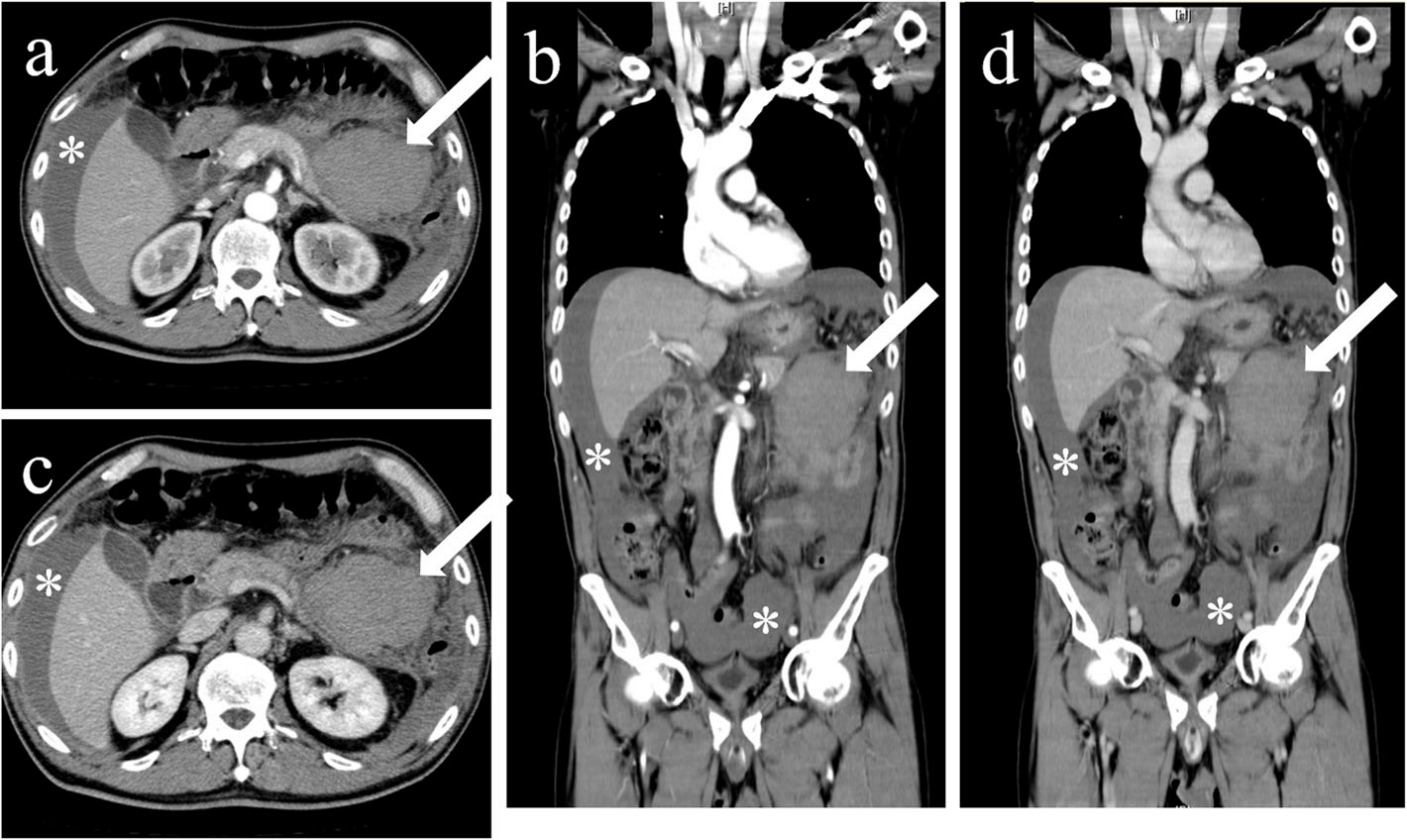
A computed tomography with contrast on arrival at our emergency department is shown; **a** an axial section and **b** a coronal section in the early phase, and **c** an axial section and **d** a coronal section in the late phase. A non-enhanced, homogenous, 8.5 cm in diameter, high-density mass lesion is located between the posterior wall of the stomach and the anterior side of the pancreatic tail (arrows). There was no sign of extravasation or aneurysmal rupture. A large amount of intraabdominal fluid collection is demonstrated (asterisks). Note that the amount of the fluid collection increased compared to the initial plain CT

**Fig. 3:**
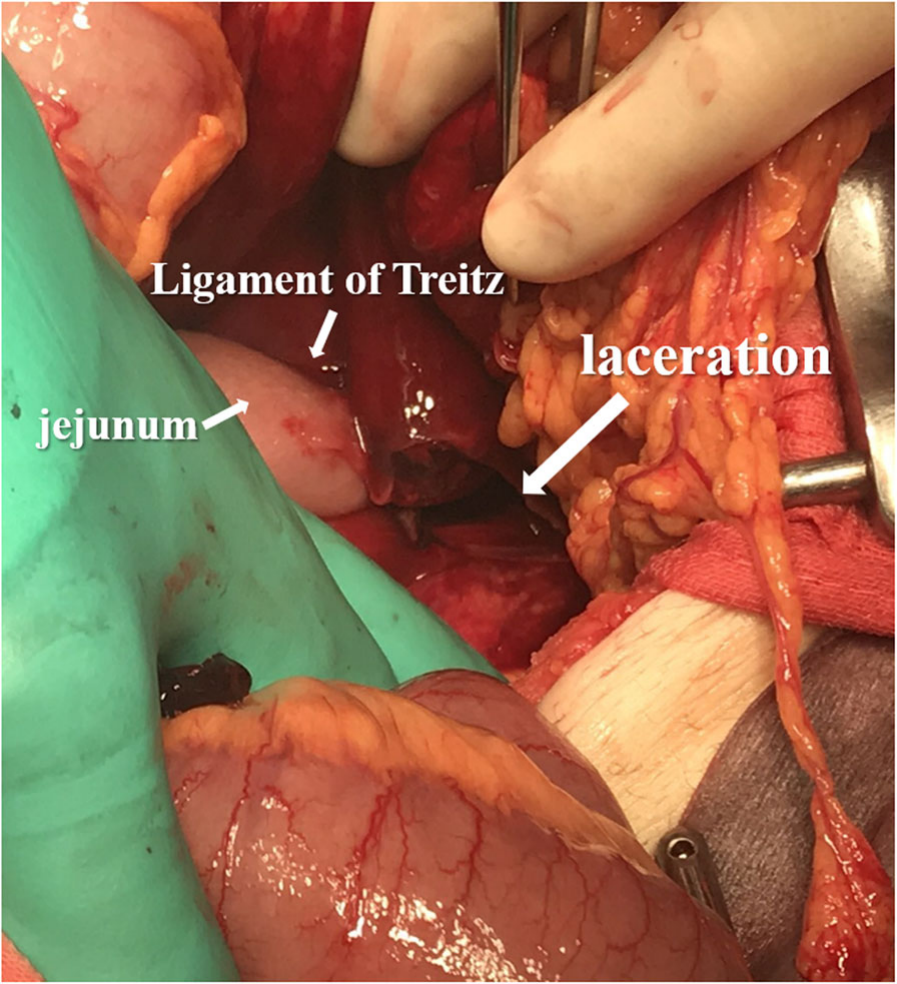
An intraoperative finding of an emergency laparotomy is shown. After removal of a fist-sized hematoma and a large amount of intraabdominal hemorrhage, approximately 5 cm in length of a mesenteric laceration in the transverse colon adjacent to the left side of Treitz’s ligament was revealed. Hemostasis was achieved by suture ligations but repair of the laceration was impossible because the tissue was too fragile

A CT with contrast demonstrated an unenhanced, high-density, homogeneous, 85 mm in diameter, mass-like lesion located to the ventral side of the pancreatic tail, and a large amount of ascites, which suggested a hematoma and massive intraabdominal hemorrhage. There was no evidence of extravasation or ruptured visceral aneurysms (Fig. 2). Considering these findings, an emergency exploratory laparotomy was performed in the operation room. The operative findings showed a large amount of intraabdominal hemorrhage and a fist-sized hematoma in the left upper abdomen. Further exploration revealed a 5 cm in length of laceration in the mesentery of transverse colon, which arose from the left margin of Treitz’s ligament extending in the left direction accompanied by oozy bleeding. Hemostasis was achieved by suture ligations with 3-0 coated vicryl; however, repair of the laceration was impossible because the tissue was too fragile (Fig. 3). Therefore, the gap of the laceration was left as it was, and absorbable hemostat materials, Surgicel Nu-Knit (Ethicon®), were applied on the oozing points. Intraoperative blood transfusion was performed with 4 units of packed red blood cells and 10 units of fresh frozen plasma. The operation was uncomplicated, and the patient was on a good postoperative course except for temporary paralytic ileus. He was discharged on the postoperative day 20. During his hospital stay after operation, we asked him about a recent abdominal trauma history. He informed us that he was suffering from intractable constipation, for which he had a habit of massaging his abdomen to facilitate defecation and he did it hard on the day before the onset of the symptom.

## Discussion

Mesenteric laceration after blunt trauma is the third common type of abdominal injury but is often accompanied by other abdominal organ injuries [1]. Isolated mesenteric laceration is very rare and theoretically seldom happens because external forces need to be conveyed directly to the mesentery in the abdominal trauma cases [5].

Chronic constipation not only often causes abdominal discomfort like abdominal bloating or crampy pain but also may give rise to serious complications such as intestinal perforation, cancer, diverticulosis, bowel obstruction, and colonic volvulus [6]. Usually, in the clinical settings, constipation is managed by dietary instructions, oral laxatives, and enemas; however, failure to pay attention to each patient’s condition may result in undesirable consequences. For instance, careless usage of enemas can cause intestinal perforation, and excessive intake of dietary fibers may lead to intestinal obstruction [7]. Meanwhile, warming or massaging the abdomen is sometimes recognized as an alleviating maneuver for chronic constipation [3]. In our patient, although his recent radiation therapy for prostate cancer might have stiffened or weakened the intestinal wall and the mesentery and damaged the autonomic nervous systems of bowel movement, the patient’s habit of massaging the abdomen by himself was surely a contributory cause of visceral organ injury.

Sudden onset of abdominal pain followed by hemodynamic instability with intra-abdominal hemorrhage instantly evokes the possibility of ruptured aortic or visceral aneurysms such as splenic, hepatic, and mesenteric arteries [8]. The initial clinical manifestation of this case was similar to that of aneurysmal rupture. Therefore, few physicians could imagine the cause of the patient’s intra-abdominal hemorrhage until the patient told us about his habits. Fortunately, due to relatively slow progress of the intraabdominal bleeding, this patient could be saved despite the delay of accurate diagnosis and appropriate treatments.

As demonstrated in this case, a history without recent trauma may confuse us to make a correct diagnosis. When the patient was referred to our hospital, the tentative diagnosis made by the previous doctor was a malignant tumor with a large amount of bloody ascites. Usually, peritonitis carcinomatosa show serous or thin bloody ascites and it is rare to see sudden hemodynamic instability in such cases. On the other hand, we could not obtain any trauma history from the patient and the patient did not show any evidence of trauma on physical examination; therefore, we did not have any idea that this was a trauma case. Primarily, we put importance on the patient’s systemic condition and findings of abdominal hemorrhage, and then we made a decision of emergency laparotomy.

Regarding the type of the operation, it may have been reasonable to perform expiratory laparoscopy, considering that he was shock in shock-grade 2 when transferred. However, we put more importance on the amount of hemorrhagic ascites and his initial hemodynamic instability at the previous hospital. He could have easily fallen in shock during or after induction of anesthesia; therefore, we think it was appropriate to proceed to laparotomy first.

In recent years, for the purpose of dieting, the habit of massaging the abdomen with hands or electronic devices has become more common, and we should remember that it could be a cause of abdominal organ injury. In addition, severe constipation should be prevented before it causes a fatal problem.

Upon considering similar mechanisms of mesenteric laceration, Heimlich maneuver for choking, rope wounds across the waist in the game of “tug of war”, and seatbelt injuries are reported as possible culprits of mesenteric injury [5, 9, 10]. The common point of these traumas including our case to cause mesenteric laceration is a rapid elevation of the intra-abdominal pressure conveyed to the stalk of the mesentery. From this standpoint, we propose that any kind of maneuver on the abdomen which may elevates intraabdominal pressure should be as much as possible avoided to prevent abdominal organ injury.

## Conclusions

This is the first report of life-threatening mesenteric injury caused by self-abdominal massage to resolve constipation, though the possibility of other etiologies such as ruptured small aneurysms could not perfectly be excluded. Abdominal massage has been reported to be effective and safe for the resolution of constipation; however, this case demonstrated that it could be detrimental. Although it is a very rare case, such complication should be reminded.

## Data Availability

The dataset supporting the conclusions of this report is included in the article.

## Abbreviation

CT: Computed tomography
